# Anatomical and Physiological Differences between Children and Adults Relevant to Traumatic Brain Injury and the Implications for Clinical Assessment and Care

**DOI:** 10.3389/fneur.2017.00685

**Published:** 2017-12-14

**Authors:** Anthony A. Figaji

**Affiliations:** ^1^Neuroscience Institute, Division of Neurosurgery, University of Cape Town, Red Cross Children’s Hospital, Rondebosch, Cape Town, South Africa

**Keywords:** children, traumatic brain injury, neurotrauma, brain, head

## Abstract

General and central nervous system anatomy and physiology in children is different to that of adults and this is relevant to traumatic brain injury (TBI) and spinal cord injury. The controversies and uncertainties in adult neurotrauma are magnified by these differences, the lack of normative data for children, the scarcity of pediatric studies, and inappropriate generalization from adult studies. Cerebral metabolism develops rapidly in the early years, driven by cortical development, synaptogenesis, and rapid myelination, followed by equally dramatic changes in baseline and stimulated cerebral blood flow. Therefore, adult values for cerebral hemodynamics do not apply to children, and children cannot be easily approached as a homogenous group, especially given the marked changes between birth and age 8. Their cranial and spinal anatomy undergoes many changes, from the presence and disappearance of the fontanels, the presence and closure of cranial sutures, the thickness and pliability of the cranium, anatomy of the vertebra, and the maturity of the cervical ligaments and muscles. Moreover, their systemic anatomy changes over time. The head is relatively large in young children, the airway is easily compromised, the chest is poorly protected, the abdominal organs are large. Physiology changes—blood volume is small by comparison, hypothermia develops easily, intracranial pressure (ICP) is lower, and blood pressure normograms are considerably different at different ages, with potentially important implications for cerebral perfusion pressure (CPP) thresholds. Mechanisms and pathologies also differ—diffuse injuries are common in accidental injury, and growing fractures, non-accidental injury and spinal cord injury without radiographic abnormality are unique to the pediatric population. Despite these clear differences and the vulnerability of children, the amount of pediatric-specific data in TBI is surprisingly weak. There are no robust guidelines for even basics aspects of care in children, such as ICP and CPP management. This is particularly alarming given that TBI is a leading cause of death in children. To address this, there is an urgent need for pediatric-specific clinical research. If this goal is to be achieved, any clinician or researcher interested in pediatric neurotrauma must be familiar with its unique pathophysiological characteristics.

## Why Children and Adults are Different

Adult physicians often underestimate the differences between adults and children. Those who work with children seldom do. Although children *are* very different from adults in physiology and disease, we commonly extrapolate data from adult traumatic brain injury (TBI) studies to pediatrics. At best this is often inappropriate; at worst it may be dangerous. The problem is that there are fewer studies in children, and so less evidence on which to base recommendations. Children are seen as a vulnerable population in ethics terms and so extrapolation from adult data is encouraged, which contributes to this practice. Its unintended consequence is weakened evidence to direct treatment for this most vulnerable population. This may be defendable if children were easier to treat than adults but unfortunately the converse is true. All of the difficulties and controversies of adult TBI are compounded in children. There are many examples. In children, the debate about thresholds for intracranial pressure (ICP) treatment are aggravated by the fact that normative values for ICP in children are not well established and depend on age. The same is true for blood pressure (BP), and so uncertainty about optimal cerebral perfusion pressure (CPP) thresholds is even greater. Resting and activated metabolic rates change across the childhood age range before settling into a reasonably stable pattern in adulthood, as does cerebral blood flow (CBF) and its response to injury. Clinical assessment is challenging—there are differences in the expected patterns of injury, clinical evaluation, imaging, and outcome assessment. Differences abound also in surgery: children have smaller blood volumes, reduced tolerance for blood loss, increased risks of long anesthesia, different reactions to medications, and reduced tissue perfusion—these are all challenging in children and so require special knowledge of TBI in childhood to optimize management. And that is not even mentioning the considerable anatomical differences. There can be little debate that children are indeed very different.

## Overview

Anatomy and physiology in children develops over several years to gradually assume the adult form. We need to be aware of these differences to prepare for common problems in childhood TBI.

## Cranial and Spinal Anatomical Differences and Implications for Treatment

**Figure 1:**
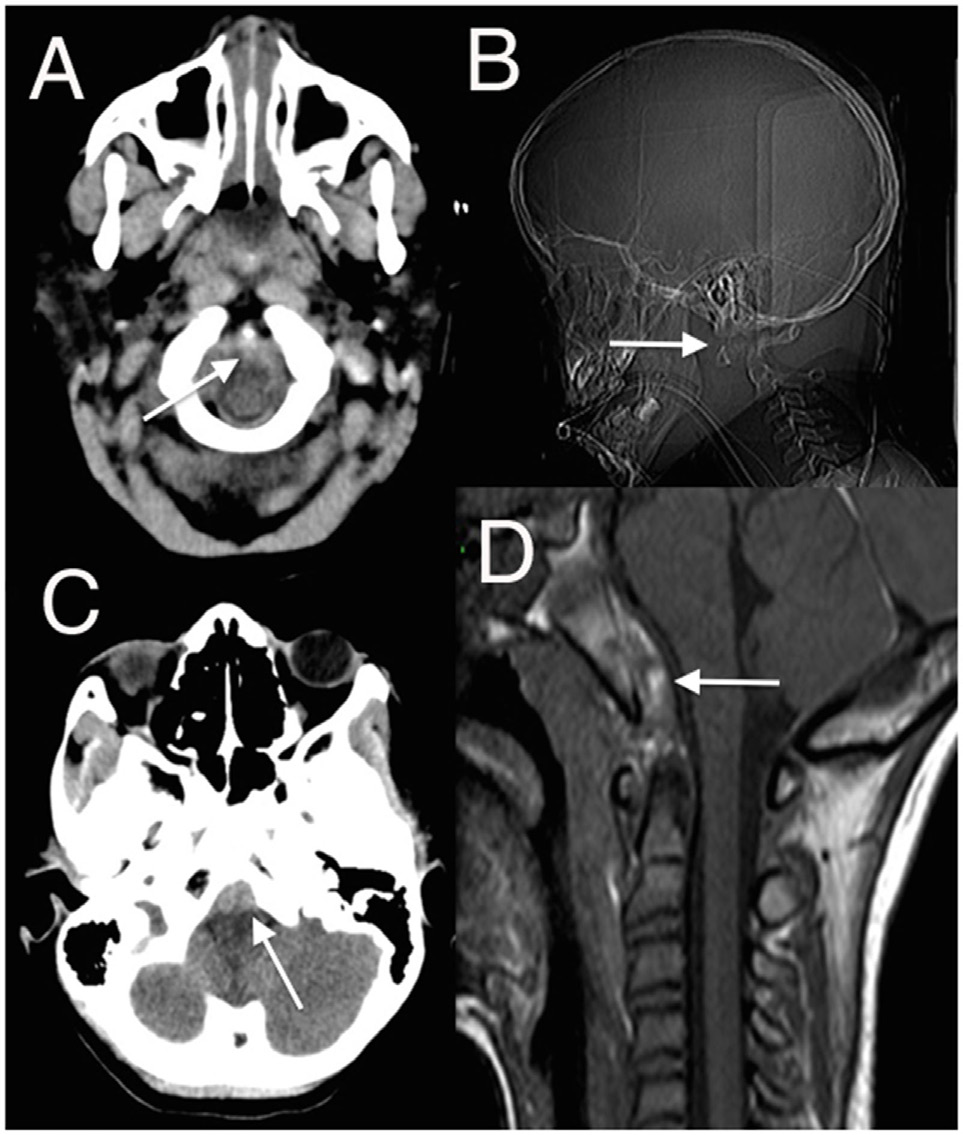
**(A,C)** Axial head computed tomography (CT) scan with low posterior fossa cuts revealing the retroclival hematomas anterior to the lower brainstem (arrowed); **(B)** CT surview showing atlanto-axial dislocation (arrowed); **(D)** sagittal T1 magnetic resonance imaging (MRI) showing retroclival hematoma (arrowed) better demonstrated on the subsequent MRI (13) (modified).

Relative to the size of a child’s body, the head is large and heavy, balanced on a neck poorly supported by weak muscles and ligaments, and so both head and cervical spine are easily injured. Biomechanical maturation of the spine is a progressive process that only starts to resemble the adult spine after age 8–9 years old. Epiphyses fuse at different times and are easily mistaken for fractures. The pattern of injuries is determined by these progressive changes. Most spine injuries in children occur in the cervical region; in younger patients, these are more often subluxations or dislocations, more often in the upper cervical spine, and more often associated with neurological injury (1–9). The fulcrum of movement descends from the upper cervical spine in young children, where C0–C2 injuries predominate, to progressively lower in the subaxial spine as they grow, when mid- to low cervical injuries become more common. The craniocervical junction is most vulnerable to injury and instability in young children because the articulations are more susceptible to movement than in older children, the ligaments and paraspinal muscles are weaker, and the dentocentral synchondrosis between the odontoid and C2 body are yet to fuse (5, 10). Congenital abnormalities of the dens and the atlas also increase susceptibility to injury. Careful attention must be paid to the lowermost axial images of the initial head computed tomography (CT), and the radiographer must ensure that craniocervical junction is well imaged: ligamentous injuries are common between C0 and C2 and are often manifest by retroclival hematomas (11, 12) (Figure 1).

In pediatric spinal injury, four patterns tend to predominate: fracture with subluxation, fracture without subluxation, subluxation without fracture [purely ligamentous injury and spinal cord injury without radiographic abnormality (SCIWORA)] (5). SCIWORA is peculiar to children and is of particular concern because, by definition, radiographs are normal (14, 15). It reflects the easy deformation of the cervical spine with external loading and the risk to underlying neural structures. Several factors cause the cervical spine in children to be weaker and thus more easily deform: weaker cervical ligaments and paraspinal muscles, increased water content of intervertebral disks, unfused epiphyses, shallow facet joints, anteriorly wedged vertebral bodies, and undeveloped uncinate processes (16–23)—these all contribute to a more malleable spine that puts neural structures at risk, even without bony injury evident on radiographs. A high index of suspicion must be maintained, and magnetic resonance imaging (MRI) should promptly be done to investigate any signs of retroclival blood or long tract findings unexplained by the head injury (Figure 1). In awake patients, five clinical criteria have a high negative predictive value for significant spinal injury: normal alertness, absence of midline cervical tenderness, no focal deficit, no intoxication, and no painful distracting injury (9).

The head and the brain are fundamentally different to adults physiologically and anatomically. In the newborn and infant, the head is disproportionally large and gradually assumes the head:body ratio of an adult over several years. Growth is particularly rapid in the first few years of life. At birth the brain is about 25% of the adult size even though body weight is about 5%; about half of the postnatal growth of the brain occurs in the first year or two; the ratio of head and neck length to body length (about 25%) in infants is almost double that of adults, and this is a continuum from gestational changes (24, 25). The disproportionally greater weight of the head also affects the movement of the head when a child falls or is struck by a moving object (26).

The skull also undergoes considerable changes with age. Fontanels and sutures close at different times. At 2 months of age, the posterior fontanel is usually closed, and by 12–18 months, the anterior fontanel is closed. Open sutures and fontanels allow some buffering of ICP, especially if intracranial volume increases slowly, but only to some extent. In trauma, intracranial volume can increase rapidly, and so the increased compliance may be rapidly exhausted. Also, normal ICP in the very young is considerably lower than in adults, as is BP, so small increases in ICP may have significant adverse effects.

The calvarium is thin in young children; this, with the sutures and fontanels, allows for easy deformation, with or without fracturing, under external pressure (27–30). Diastatic skull fractures may also occur in children (31), where an unfused suture diastases as a result of direct trauma or deformation, and sometimes with raised ICP. Because of the pliability of the skull, a linear fracture may represent significant underlying parenchymal injury sustained by marked deformation at the time of injury despite little evidence on the head CT. This makes growing skull fractures a unique feature of young children (32–35). At the moment of impact, the deformed bone and fractured edges tear the dura. Soft tissue interposes between the fractured edges which then do not heal. The pulsatility of the brain and the growth of the cranium then combine to increase the fracture size over time, which further retracts the dural edges in a vicious cycle. So, surveillance for growing fractures is important and these require surgery. On the other hand, closed depressed, “ping-pong,” fractures are common in very young children and can often be treated conservatively. They often mold to normality over a few months.

The thin skull may be a challenge for ICP monitoring—often surgeons are reluctant to use bolt systems or even measure ICP in the very young (36). If bolt systems are used in young children, the skull thickness must be measured on the head CT and the bolt thread adapted accordingly. Alternatively, the monitor can be tunneled. The young age of a patient should not be a reason not to monitor ICP.

If the dura is intact, small skull defects often heal well due to the osteogenic potential in childhood bone; however, resorption rates after bone flap replacement are higher in young children (37), especially when there is a significant delay in the bone being replaced. The growing head size and pulsatile nature of the brain contribute not only to this risk but also to the problems of cranioplasty using foreign material (37, 38). Split calvarial grafts are ideal in this situation but unfortunately the underdeveloped medullary layer makes this difficult in children under the age of 3.

Basal skull fractures are common, especially in crush injuries, in which release fractures may occur diagonally across the skull base. These must raise suspicion of an injury to the carotid artery (39) in the canal, especially when running into the sphenoid bone, and may warrant MR angiography. The vessel may be occluded by dissection and/or thrombus and this may be clinically silent in children if the crossflow through the circle of Willis is adequate. Even if the occlusion is asymptomatic, it is important to diagnose this because of the potential for thrombus extension.

At the mild end of the spectrum, decision-making about head CT in children is compounded by the greater sensitivity of the developing brain to the effects of radiation, in terms of both cancer-inducing potential and cognitive development. Therefore, several sets of decision rules have been evaluated to rationalize the indications for head CT to limit over-investigation (40). Even if resources were not a problem, the solution is not as straightforward as lowering the threshold for MRI. Children under the age of 8 years old usually need sedation or general anesthesia for MRI, which carries relatively low risk, but risk nevertheless. Also, there is growing concern of the effects of long and cumulative anesthetics on the developing brain, although this remains controversial (41).

The radiological pattern of pediatric TBI shows some differences to that of adult TBI (42). In severe pediatric TBI, diffuse injuries are more common than the focal injuries and contusions of adult TBI. In diffuse injuries, the scan looks relatively benign but the patient is in deep coma. A contusion in the midbrain is not uncommon as a manifestation of significant injury that may be subtle on head CT; MRI demonstrates the lesion more clearly. Patterns of injury are also determined by the mechanical properties of the brain tissue, which in children is stiffer than in adults (30). Non-accidental injury (“shaken baby syndrome”) is peculiar to young children (43, 44) and is beyond the scope of this article but is an important specific pathophysiological entity to be aware of. The pathophysiology, radiology, clinical presentation, and outcome are very different to accidental injury in many ways, and this requires separate consideration.

**Figure 2:**
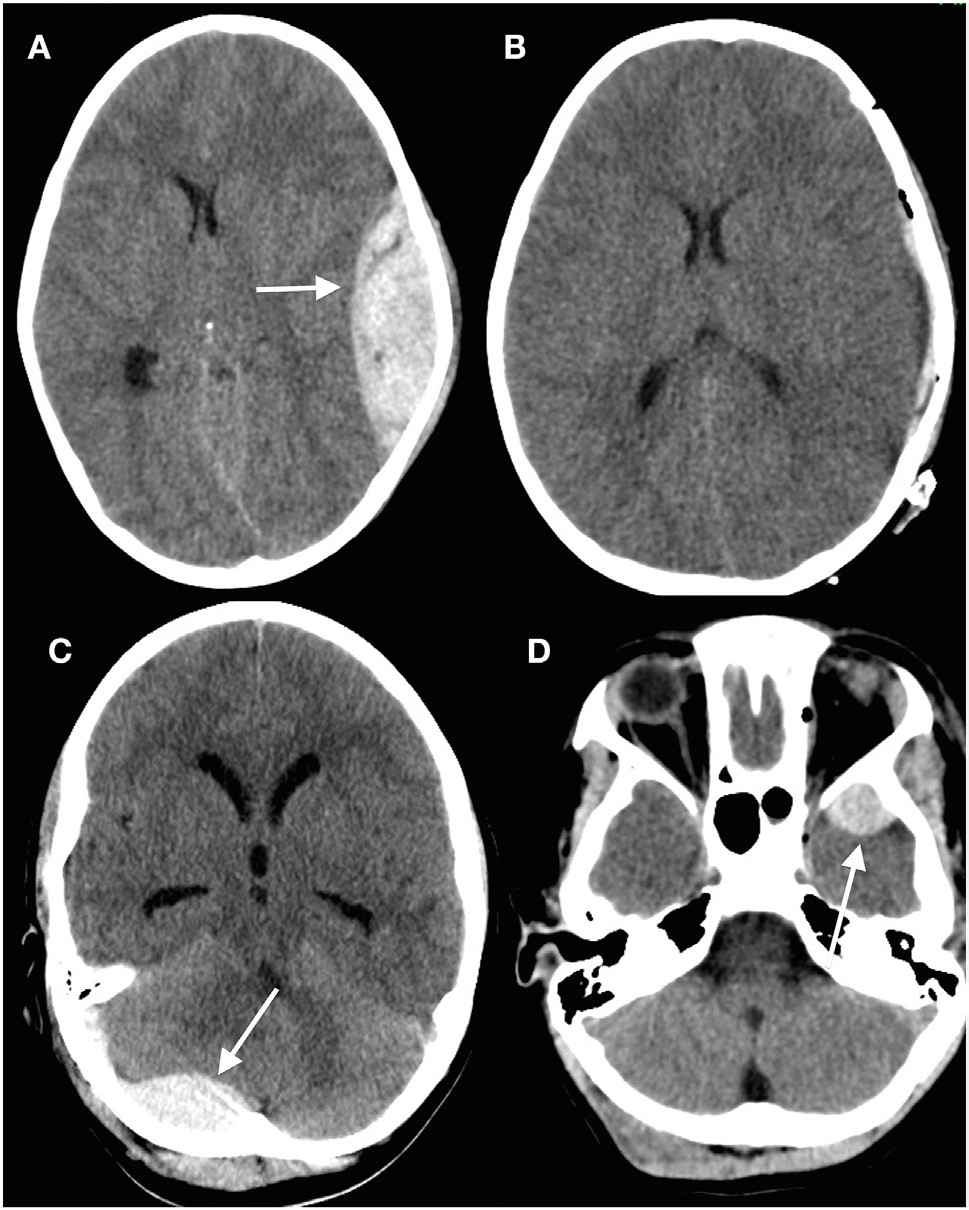
Epidural hematomas occur in a variety of locations. **(A)** Head computed tomography (CT) showing a typical convexity epidural hematoma in a child; **(B)** evacuated hematoma in the same patient; **(C)** posterior fossa epidural hematoma (arrowed) underlying a suboccipital fracture; **(D)** epidural hematoma anterior to the left temporal tip (arrowed) (13) (modified).

**Figure 3:**
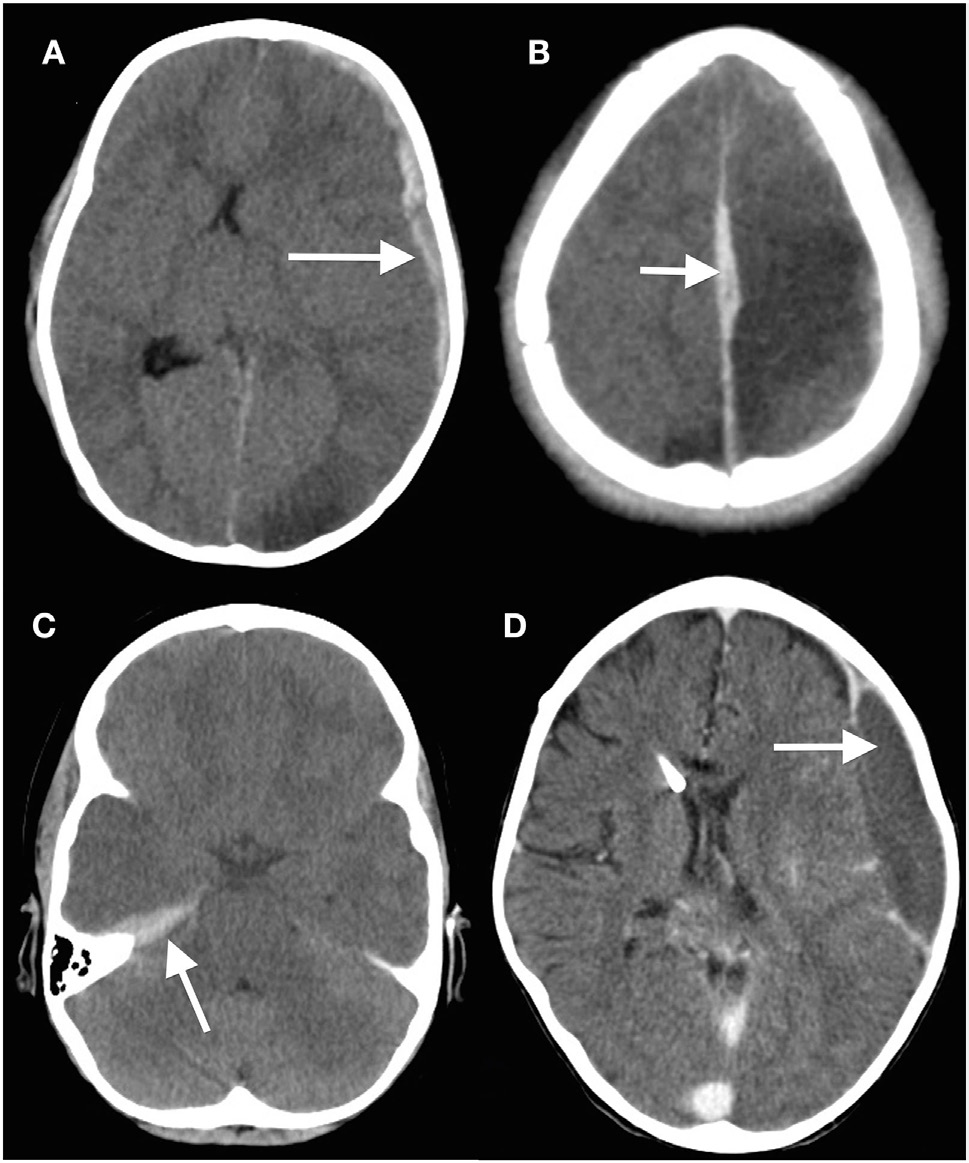
Subdural hematomas in children. **(A)** Head computed tomography (CT) scan showing a typical acute subdural hematoma with a hypodensity in the ipsilateral posterior cerebral artery territory; **(B)** interhemispheric subdural hematoma (arrowed) with adjacent venous hypodensity; **(C)** subtle subdural hematoma situated on the tentorium beneath the temporal lobe; **(D)** minor knocks to the head can easily cause a subdural hematoma (arrowed) in children with ventriculoperitoneal shunts, especially if there is a degree of overdrainage from the shunt (13) (modified).

Discrete hematomas in children are less common than in adults but of course do occur. Epidural hematomas in children are somewhat different to those in adults (45–47). The middle meningeal artery is not as incorporated into bone as in adults, but epidural bleeds from the edges of a fracture easily lead to hematomas. They occur in a wider variety of locations (Figure 2), in part because these are often due to venous rather than arterial hemorrhages (13). Fractures in the occipital and suboccipital regions are particularly concerning because of the risk of a posterior fossa hematoma, which rapidly causes brainstem compression as well as hydrocephalus by fourth ventricular and aqueduct obstruction (48–50). Subdural hematomas (Figure 3) are associated with more severe injuries to the parenchyma, cortical veins, and venous sinuses (51–53). Associated arterial and venous infarcts are not uncommon. Non-accidental injury must also be considered in infants where there are bilateral subdural collections, particularly of differing ages (28, 54); however, one must also keep in mind that there are other medical and procedure-related causes of subdural hematoma (53, 55–58).

## Systemic Issues and Implications for Treatment

Several systemic anatomic and physiologic differences are relevant when managing head trauma in children. Young children are at particularly high risk of airway obstruction. Their tongues are relatively large for their oral cavity, as are the soft palate and soft tissues of the mouth and the epiglottis, which is relatively longer and stiff. They also have a larynx that is higher and more anterior, a cricoid ring that represents the narrowest point of the airway, and a shorter trachea that bifurcates higher (59, 60). The trachea has a small diameter and is compressible, so even small changes in diameter or foreign bodies can rapidly lead to airway compromise; frank respiratory embarrassment is accelerated by their reduced functional residual capacity and higher metabolic requirements per weight (61). Because they have a large occiput, their necks flex easily when lying supine, which may contribute to airway compromise. The chest wall is cartilaginous and more easily deformable; rib fractures are unusual but lung contusions are common and may be severe despite little external evidence of injury (61, 62). Because of their small lung volumes it is easy to unintentionally hyperventilate children during resuscitation (especially with manual ventilation) and so hypocapnea is common, which may be particularly detrimental at a time when CBF is already reduced. Abdominal injury and gastric distension easily constrain breathing because children are diaphragmatic and abdominal breathers.

Insensible fluid losses and heat loss is common, and so hypothermia easily occurs, especially in the very young: neonates and infants have body surfaces as much as three times that of an adult, with proportionally large heads for their body size. Bones break or are deformed easily and so polytrauma is common—long bone fractures, chest wall injuries, and injuries to underlying intrathoracic and intra-abdominal organs. Rapid low dose whole body radiographs may reduce the overall radiation burden in children who have suspected polytrauma and are a useful rapid screening tool (63). Injury to solid organs is relatively common because children have proportionally larger organs (which are also closer to each other), less intraperitoneal fat and weaker abdominal musculature as protection (62, 64, 65). Focused abdominal ultrasound has a high specificity for detecting hemoperitoneum (66). Fortunately, most abdominal injuries in children can be treated conservatively (62, 67).

Blood pressure control is pivotal both in the intensive care unit and in the operating room. During surgery, anesthesiologists often maintain relatively low BPs to reduce blood loss. However, this may compromise perfusion, both in handled tissues and a swollen brain. Given the importance of BP control in surgery, there is surprising variability in how hypotension is defined. For some, it is a decrease of more than 20–30% from the baseline systolic blood pressure (SBP), others use variable normograms (68). For neurosurgical patients though, we need to maintain perfusion not only of physiologically normal tissue but also of tissues penumbral to a lesion. Hypotension tolerable in normal children may cause harm in children with TBI. At the same time, high BPs cause unnecessary bleeding, as well as brain swelling if autoregulation is impaired.

Hypotension must be avoided in TBI—there is a similar association between hypotension and poor outcomes in childhood TBI as in adult TBI (69). But there are several additional challenges: as stated above, BP normograms are often not used, and the circulating blood volume changes dramatically with age (70, 71). In young children, this is a small volume—“minor” blood losses can have major clinical implications. Neonates have a circulating blood volume of approximately 85–90 ml/kg, infants 75–80 ml/kg, older children 70–75 ml/kg, and adults 65–70 ml/kg. Therefore, loss of 50 ml in a 3 kg child represents almost 20% of their circulating blood volume. The issue of BP maintenance is discussed further below.

## Brain Physiology and Monitoring

### Cerebral Compliance

Open fontanels and unfused sutures allow for increased cerebral compliance in young children, but only to a point. When intracranial volume increases rapidly, as in trauma, raised ICP is as important an issue in young children as it is in older children and adults, perhaps even more so because of the low normal range of ICP in this age group. Still, ICP monitoring is used infrequently in these children (36). Cerebral compliance is also affected by CBF and volume, and the ratio of cerebrospinal fluid (CSF) volume to brain, all of which are age-dependent.

**Figure 4:**
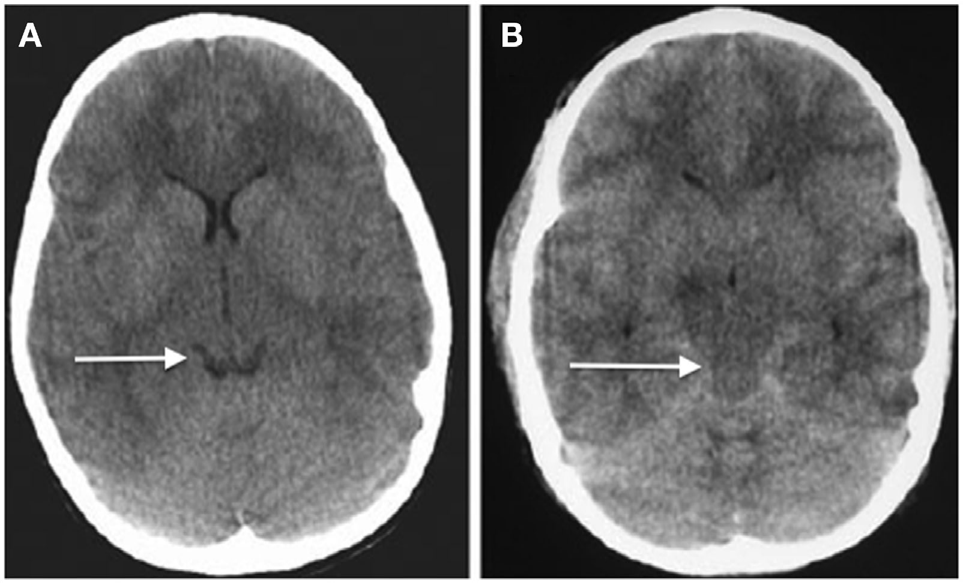
Axial head computed tomography (CT) scans showing **(A)** relatively normal looking hemispheres with open basal cisterns (arrowed, “smiling brain”) and **(B)** diffuse severe swelling and obliterations of the cisternal spaces (arrowed) (74) (modified).

Given the differences in CSF-brain ratios, clinicians must be familiar with typical imaging across the age ranges—it is easy to misinterpret the likelihood of raised ICP because of these differences. The CSF-brain ratio reflects the balance between brain tissue and CSF in the ventricles and subarachnoid cisterns of the brain. Although this has not been formally quantified across the age range, radiologists and pediatric specialists are aware of the differences between very young children, older children, and adults with respect to the amount of intracranial CSF that is expected, reflecting the growth of the brain from the neonatal stage through childhood and the development of atrophy with age in adults (72, 73). As in adults, the patency of basal cisterns is an important indicator of ICP (Figure 4), but it is by no means absolute (74). Just because the cisterns are open does not guarantee that ICP is normal. Also, brain swelling can change rapidly over time, especially in children, in whom cerebral blood volume changes are a common cause of increased ICP. Therefore, what the scan looks like at one point in time may bear little semblance to what it looks like several hours later.

### Cerebral Blood Flow

Understanding CBF is more challenging in pediatric TBI, in part because hyperemia is reported to be a frequent cause of raised ICP (75) and because normal CBF varies with age. These changes with age are probably the reason that diagnosing hyperemia in children is not as straightforward as it would seem (76–78), so it would be easy to misinterpret what may well be normal for a particular age group. It is also difficult to study; the data we have currently heavily depend on the tools used to determine CBF. These have included ultrasound-based techniques, MRI, and positron emission tomography (PET). The conditions under which the study is performed also markedly affect the outcome. Studies are difficult in children and so sedation or anesthesia is often used, both of which of course affect CBF. To illustrate, across three studies in children, using different techniques, the following results are reported for average total CBF: 760/781 ml/min (girls/boys, respectively), 1,101 ml/min (girls and boys), and 1,538 ml/min (girls and boys) (79–81).

Still, there are a few things we know. Rapid changes in metabolic demand in the early years follow cortical development, progressive myelination, and synaptogenesis. CBF is lowest at birth and in neonates, peaks at ages 3–7, and then progressively decreases to adult levels (78, 80–83). CBF volume shows similar changes. The sharpest increase is in the first 6 months of life; this continues over the next 3 years at a slower pace. In 3-year olds, the CBF volume is ten times greater than in the newborn (84). CBF volume in neonates is 70 ml/min and about 700 ml/min in 3-year-old children (80). In a PET study of children, regional CBF was 140–175% of adult values for children between the ages of 3–7 years, although cerebral metabolic rates of oxygen were less markedly different (100–120% of adult values) (83). When normalized for brain volume, which of course changes with age, global cerebral perfusion (total CBF divided by brain volume) reaches a peak of around 2.5 times that of adults between the ages 3 and 4 (81, 85).

But it is not just the baseline differences that matter. Pediatric brain metabolism also responds differently to activation. One study of 8–12-year-old children showed a similar percentage increase in CBF after activation but a greater increase in *absolute* flow when compared with adults (86). This may account for some of the dynamic changes in ICP in children with TBI despite minimal stimulus. Cardiovascular changes with age must also be considered. A higher metabolic rate in children is associated with higher cerebral and cardiac indices. A greater proportion of the cardiac output goes to the brain in children, in keeping with the higher cerebral metabolic rate—the fraction of cardiac output to the brain is more than twice that of adults (81). All of these factors affect the hemodynamic changes in ICP and CPP in children with TBI.

So, when determining what CBF is in normal and pathological states, whether ischemic or hyperemic, it is clear that several age-related phenomena must be considered. Unfortunately, the evidence base is lacking because children are inherently more difficult to study than adults, and there are few bedside tools that can be applied to children with TBI that provide good data. Transcranial Doppler (TCD) is an example of such a tool. It is commonly used but has significant limitations, in particular because only flow velocity in the basal vessels is determined. High flow velocity must be distinguished from what is normal in children based on age and what may be vasospasm. Little is written about posttraumatic vasospasm in children and unfortunately the Lindegaard ratio is not always reported, an important factor to consider when interpreting high cerebral blood velocities values by TCD (87, 88). This is the ratio between the flow velocity in the middle cerebral artery over the flow velocity in the internal carotid artery. In adult patients, it is reported that to diagnose vasospasm the flow velocity in the middle cerebral artery should be greater than 120 cm/s, and the ratio should be greater than 3 to diagnose vasospasm. To date though, this has not been validated in children. In general though, vasospasm appears to be less common in severe pediatric TBI than in adults (89); however, one study reported vasospasm in as much as one third of children (90). These figures are based on TCD parameters developed in adult subarachnoid hemorrhage patients and so may not apply to children. Much work needs to be done using bedside tools to guide hemodynamic and metabolic changes by the bedside.

### Intracranial Pressure

**Figure 5:**
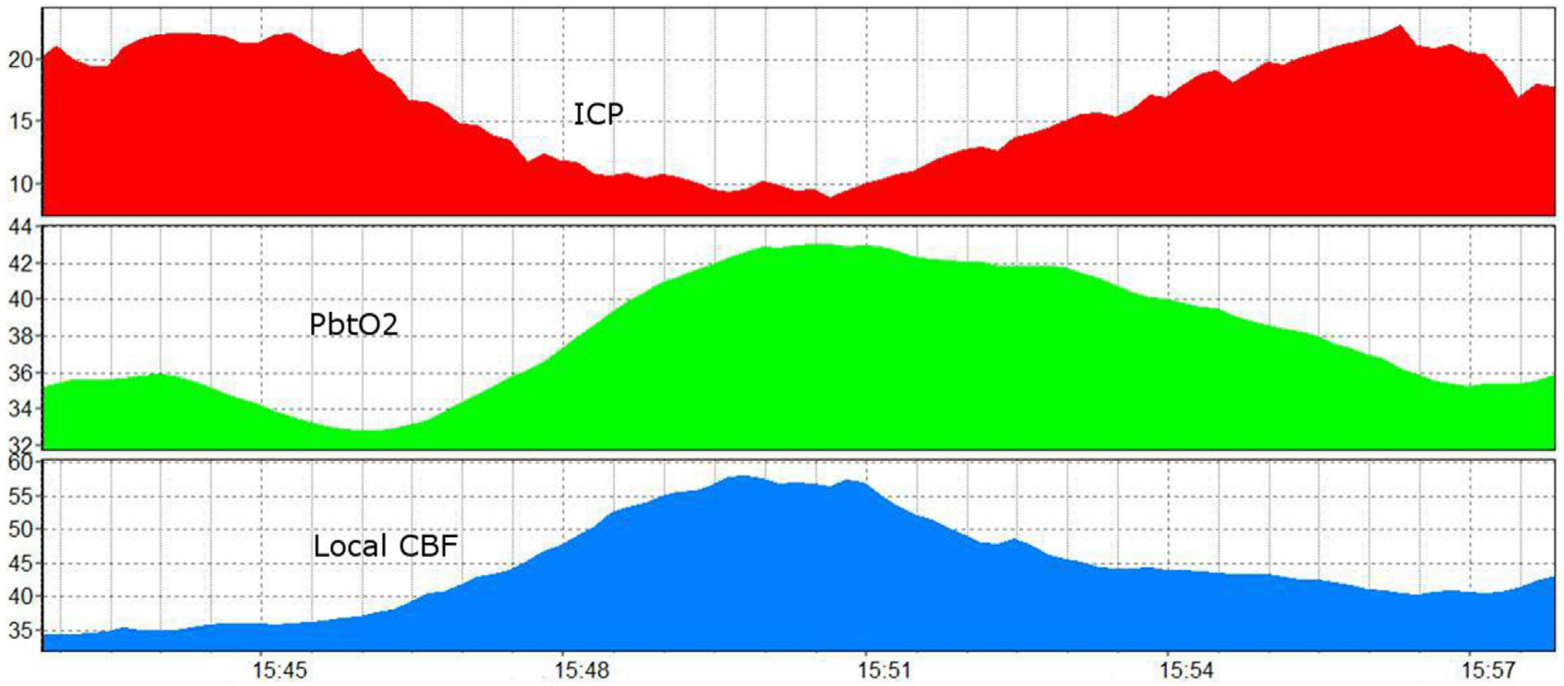
A 15 min recording showing the influence of increased ICP and ICP reduction on brain oxygenation and brain perfusion (92) (modified). ICP, intracranial pressure (red, in mmHg); PbtO_2_, brain tissue oxygen (green, Licox, in mmHg); CBF, local cerebral blood flow (blue, Hemedex local tissue monitoring, in ml/100 g/min).

Although it is clear that ICP is injurious to the brain as a secondary mechanism, causing brain shift and brain ischemia, with often an inverse relationship with perfusion of the brain (Figure 5), there is ongoing controversy about ICP thresholds for treatment in adult patients, recently aggravated by the South American trial of ICP monitoring in severe TBI. Although the trial has been criticized and it is currently recommended that existing protocols for ICP monitoring should not be changed (91), there is no doubt that greater uncertainty has crept into the management of raised ICP in trauma. Arguably, this trial may have been unsuccessful because there was a singular focus on ICP, with little consideration given to the complexity of cerebral dynamics. There are several different causes of increased ICP but recommended treatment protocols are insensitive to these. Therefore, ICP treatments may well be inappropriately applied. Furthermore, given the heterogeneity of causes of increased ICP as well as inter-individual differences, ICP thresholds for injury likely vary across patients. Lastly, all ICP therapies have adverse consequences and so the risk-benefit ratio should ideally be determined for each situation.

These are some of the obvious controversies and uncertainties in managing ICP in adult TBI. To this is brought further unknowns for pediatric TBI. Much of the pediatric recommendations for TBI care are extrapolated from adult studies; there is very little pediatric-specific evidence. This is why the recommended ICP treatment threshold is also 20 mmHg (93), despite general awareness that normal ICP is lower in children and the fact that raised ICP in children behaves differently. To date, there are no age- or cause-specific recommendations for thresholds or therapies.

A key starting point is knowledge of normative ICP values in children, for which there are surprisingly little data. Much of the existing knowledge derives from examination of CSF opening pressures from lumbar punctures. A series of these studies from one institution (94–97) sought to determine ICP thresholds in children. They examined children aged 1–18 years old who apparently had no condition that would increase ICP: 1,066 children were screened, the authors enrolled 472, and after exclusions investigated 197. Their results suggested that the upper limit of normal ICP for children was 28 cm H_2_O (20.6 mmHg). The opening pressures were normally distributed and showed a mean of 19.6 cm H_2_O, with 10th and 90th centiles of 11.5 cm H_2_O and 28 cm H_2_O, respectively. The authors then went on to study children who had fundoscopic evidence of optic nerve head edema and reported their results in keeping with their previous findings.

Cerebrospinal fluid opening pressures are commonly used as a measure of ICP, ever since it was first described in 1891 by Quincke (98). Still, there is no general agreement that it accurately reflects ICP, especially in diseased states. Cartwright et al. studied 12 children (mean age 8.5 years) who were being monitored with an intracranial device (Camino, Integra Neurosciences) and who underwent lumbar puncture. The values were quite discrepant (*p* < 0.001): mean ICP from the Camino monitor was 7.8 mmHg compared to 22.4 mmHg from the lumbar puncture. The authors suggested that lumbar CSF opening pressures significantly overestimated the true ICP (99). The lumbar puncture technique was similar to that used elsewhere. The case mix included patients referred for evaluation of craniosynostosis and idiopathic intracranial hypertension (there were no TBI patients). This affects the generalizability of their results, but such is true also for studies of “normal” children—we generalize physiology to pathological conditions. Lumbar opening pressures are affected by several variables—including technical factors such as patient positioning and sedation, as well as pathology-related factors. Furthermore, a once-off determination of ICP does not reflect compliance, dynamic ICP changes, or ICP behavior after stimulation. Lastly, the ICP tolerated in the normal physiological state may not be tolerable if there are already factors reducing tissue perfusion or if physiological mechanisms such as pressure autoregulation and flow-metabolism coupling are impaired.

So even if these numbers are accurate for normal children, can they be applied to a swollen, ischemic brain? At what point is brain perfusion impaired? Is the cause of increased ICP relevant to this decision? Accumulating evidence suggests that the relationship between ICP and perfusion of the brain is complex (92). In children, ICP often changes rapidly from 1 min to the next. Often, the cause of this appears to be vascular in nature. In keeping with this, simultaneous increases in ICP and brain oxygenation (or CBF) are often observed, but only to the point where the rise in ICP (presumably from increased cerebral blood volume) appears to have an adverse effect on tissue perfusion, at which point the relationship changes. The point at which this happens is variable and there may not be a specific threshold consistent across all children. This phenomenon, along with age- and cause-specific differences, produces heterogeneity and explains the observation that the relationship between ICP and brain oxygenation is weak when pooled across all patients, even though they may be tightly linked in episodes in individual patients (92). Indeed, this may represent part of the interindividual variability that confounds many of our treatments and leads to negative studies, in large part because not all patients respond the same to treatments, or in fact need that particular treatment at all (100). This raises several questions: should the threshold for ICP treatment be different if the cause of increased ICP is increased blood flow, i.e., if perfusion is not compromised can we be permissive about ICP higher than our traditional target? Conversely, if perfusion is affected at ICP thresholds less than 20 mmHg, should we intervene earlier? These have been some of the questions driving the use of multimodality monitoring to make individualized, or at least better, decisions at the bedside (101).

### BP and CPP

If determining ICP treatment thresholds in children is complicated, attempting the same for CPP is worse. CPP depends on ICP and mean arterial pressure (MAP), and so to the uncertainty around ICP are added the variables about changing normative BP across the age range and the complexities of pressure autoregulation. It would appear reasonable that adequate CPP should be age-based but no such recommendation exists. For the ideal normative BP range, sex and height should also be considered but rarely are. Even the definition of hypotension is surprisingly variable (see above). Most definitions rely on SBP, not MAP. Various estimations based on age exist, particularly so for resuscitation. One common estimation for SBP at the 5th centile (at 50th centile for height) is 2× age in years + 65; for MAP this is adjusted to 1.5× age in years + 40. The 50th centile for systolic BP is calculated as 2× age in years + 85; and for MAP, 1.5× age in years + 55 (102). The Pediatric Advanced Life Support guideline is slightly different: hypotension is defined as SBP less than the following thresholds: 60 mmHg for neonates, 70 mmHg for infants (1–12 months), (2× age in years + 70) for children aged 1–10, and 90 mmHg over the age of 10. Useful tables can be found from an analysis of data from 60,000 children in the National Center for Health Statistics database (102), in which the authors charted 5th–95th centiles accounting for age, height, and sex. They also compared their definitions of hypotension to that of other sources (102).

These recommendations about BP management are for generally ill patients, not for those with a brain injury, for whom optimal BP control may be substantially different. To start, MAP is more useful than SBP and ICP must be known to calculate CPP. Some institutions prioritize CPP above ICP, arguing that CPP is the ultimate driving force for perfusion of the brain. This may be so but increased tissue pressure can decrease local tissue perfusion regardless of CPP and impaired autoregulation may exacerbate the risks of chasing a target CPP.

Adult practice in various centers has ranged from aggressive CPP management (103) to minimized CPP targets (104). What is clear is that chasing higher CPP targets increases the risk of lung pathology due to aggressive fluid and inotrope administration (105). There is a growing consensus that an optimal CPP varies substantially between patients. But how best to optimize CPP individually remains uncertain. One school of thought argues that an optimal CPP can be determined from passive correlation analysis between BP and ICP (as a proxy of blood volume) and in so doing develop a pressure reactivity index, which allows calculation of an “optimal CPP” target at which autoregulation is most active (106). However, just because the CPP is optimal with respect to that measure, it does not necessarily follow that a patient needs that CPP for adequate brain perfusion. Others use various ancillary measures to determine the adequacy of blood flow to the brain such as microdialysis and brain oxygenation.

The published guidelines for children suggest a CPP threshold of 50 mmHg in older children and 45 mmHg below the age of 2 (93), but the evidence base for this is weak. No age-based recommendations exist for children. Autoregulatory status likely affects an optimal CPP, but this is rarely used (see below). Similarly, measures of brain perfusion adequacy are also rarely used (see below). Importantly, current data suggest that patients managed according to the published guidelines still commonly (around one third) experience episodes of very low brain oxygenation despite adequate adherence to targets for ICP, CPP, and systemic oxygenation (107).

There is also ongoing discussion about whether MAP should be zeroed at the level of the head or the heart. This problem is arguably exacerbated in children because the difference in vertical height between the head and the heart when patients are managed with elevation of the head of the bed is more variable in children because it is influenced by the length of the patient. However, some argue that these differences are of little clinical consequence because perfusion of the brain is subject to the siphon effect—it is in essence part of a closed loop of perfusion from the heart to the brain and drainage back to the heart (108, 109).

### Autoregulation

About 30–40% of children with severe TBI develop impaired autoregulation in the acute setting (110–112). When autoregulation is impaired patients are at greater risk at both lower and upper ranges of BP. Therefore, it is unsurprising that impaired autoregulation is associated with worse outcomes (113). Currently, there are no recommendations of how autoregulatory capacity should be considered in the management of pediatric TBI.

Few studies have examined autoregulation in pediatric TBI, either as dynamic testing or pressure reactivity index estimation (110–118). It remains uncommon that autoregulation is measured as part of clinical care in pediatric TBI, despite the impact this has on the relationship between BP and cerebral hemodynamics, including cerebral blood volume (and therefore ICP) and cerebral perfusion. It would be sensible to have a measure of autoregulatory capacity to assist decision-making. BP can be titrated against some measure of perfusion adequacy while aware of its influence on cerebral blood volume, such as combining brain tissue oxygen (PbtO_2_) and ICP monitoring. If autoregulation is impaired, augmenting BP to achieve CPP simply increases ICP. At best it is of little help, at worst it may be detrimental. Therapy should be focused on ICP reduction. If autoregulation is intact, there is greater capacity for BP augmentation to benefit CPP. In some patients, this may actually result in some reduction of ICP because of the vasoconstrictive response.

### Carbon Dioxide Reactivity

Carbon dioxide (CO_2_) reactivity is a robust and well described response of cerebral arterioles that is usually preserved in the injured brain (119). Occasionally, it may be impaired in the first few days after injury (120). The mechanism is of great clinical relevance because of therapeutic potential as well as unintended changes in CO_2_. We have to be particularly aware of this in children, where the mechanism appears stronger than in adults (78). Unintended changes in CO_2_ are particularly common in children because of their small lung volumes—for this reason, accidental hyperventilation is common during resuscitation. Because hypocapnea vasconstricts cerebral arterioles, it decreases cerebral blood volume and therefore ICP, but at the cost usually of decreased CBF (in the normal physiological state anyway). This is particularly hazardous in the early phase of head injury, where CBF may be abnormally low. Conversely, a rise in CO_2_ vasodilates cerebral arterioles, which may lead to increased perfusion, but also increases cerebral blood volume and therefore ICP. The subsequent increase in ICP may have a secondary negative effect on perfusion. This paradoxical effect may also be seen in hyperventilated patients with high ICP. Despite the fact that hypocapnea decreases arteriolar diameter, occasionally the reduction in ICP has a net beneficial effect on perfusion, at least until a certain threshold is reached. Beyond that, the effect likely reverts to expected reduction of perfusion associated with vasoconstriction as there is no further perfusion benefit of reducing ICP below that threshold. The key is not to assume how CO_2_ will affect perfusion, but preferably to measure it.

In the past, hyperventilation was recommended therapy for raised ICP before a randomized controlled trial reined in this enthusiasm (121). The specifics of that trial, though, are worth reconsidering. Most importantly, it examined a very specific application of hyperventilation, namely prolonged, severe, and not targeted to a specific ICP crisis. The treated group was hyperventilated to a mean arterial CO_2_ of 25 mmHg; they fared worse at 3 and 6 months, but no differently at 12 months. The practice of hyperventilation declined after this trial, but it is still considered an option in current guidelines, under some sort of perfusion monitoring. Unfortunately, this has not been tested in large populations. The practice of hyperventilation though (uncontrolled by perfusion monitoring) is still common—in the controlled trial of ICP monitoring referenced above, hyperventilation was used in 60% and 73% of the ICP monitoring group and imaging-clinical groups, respectively. Manipulation of CO_2_ in a controlled environment may still be of value, but we need to examine its use for more limited time periods, controlled with perfusion monitoring, and as a strategy to break an ICP crisis. However CO_2_ manipulation is used though, it must be remembered that the effects are temporary.

### Brain Oxygenation Monitoring in Children

Methods of brain oxygen monitoring (122) have not been extensively studied in children. Of the various methods, invasive tissue monitoring (PbtO2, or partial pressure of brain tissue oxygen) and near-infrared spectroscopy (NIRS) have some data in pediatric TBI. NIRS has been evaluated more commonly in neonates and cardiac patients; there are fewer studies relevant to TBI. There are currently more studies of PbtO2 (107, 110, 123–128); although the number remains small compared to those in adults. Data from the largest series (126) is consistent with the adult experience in terms of thresholds related to outcome, although one report suggested a higher threshold may be more predictive for a favorable outcome (128). Published data and clinical experience also confirm its value as an ancillary test in patients diagnosed with brain death and as predictors of mortality and functional outcome (123, 126, 129). Whether treatment directed at maintaining PbtO2 improves outcomes is yet to be determined in adults and children.

Importantly, the relationship between PbtO2 and ICP is mixed. Although in some patients, there is often a clear and strong negative correlation, when averaged over several patients, the correlation is poor (92) for several reasons—variations in autoregulation, different responses to CO_2_ changes (as discussed above), hyperemia, vasospasm, and electrophysiological events—all of which create complex relationships between ICP and brain perfusion.

### CBF Monitoring

Cerebral blood flow monitoring is not used very often in pediatric TBI, in large part because the tools are not well developed (130). Spatially resolved techniques are of limited application because of the dynamic nature of pediatric cerebral hemodynamics. Local CBF monitoring shows good temporal resolution but poor spatial resolution, which is true for all forms of catheter based monitoring. The most frequent reports in children involve TCD recordings, which of course measure flow velocity in the basal vessels of the Circle of Willis, not true flow. Still, it has applications that may be of use, including as a tool to determine flow changes in autoregulation tests, detect vasospasm and perhaps as a non-invasive measure of ICP (131). Other limitations of TCD are that it is operator dependent and long-term monitoring is difficult because changes in the insonation angle affect recorded values. O’Brien has published reference values for critically ill and sedated children (132).

The Bowman perfusion monitor (Hemedex) uses a thermodilution method to determine local CBF, but it has not been widely used (133). Imaging of blood flow varies from perfusion CT to PET imaging. These may be valuable in research and for point-in-time assessments of brain perfusion (excellent spatial resolution) but are less helpful for managing the dynamic nature of brain injury (poor temporal resolution). Brain physiology is dynamic in the acute phase and responds differently over time due to changing systemic physiology. Because of radiation concerns, xenon CT is rarely used, but previous studies produced some insights. Adelson et al. studied CBF in 95 children with xenon CT and found that unfavorable outcomes were associated with reduced mean CBF (134). When CBF was less than 20 ml/100 g/min in the first two days postinjury, outcome was universally poor. Disturbed CO_2_ vasoreactivity was also associated with poor outcomes.

### Brain Metabolism

Brain metabolism in children changes with advancing age. It depends on progressive myelination and synaptogenesis and drives the substantial changes in CBF, especially in the first 8 years of life (83, 84). Cerebral metabolism of glucose starts at low rates of around 60% of adults values at birth, but rapidly accelerates to over 200% adult values by age 5 before slowly decreasing to adult levels through adolescence (135). As yet, it is unclear what implications this has for treatment, including the most basic aspect of supportive care, nutrition. Currently, there are no clear recommendations on when and how to feed after severe TBI in children (93), and current practice variation across centers is wide (136).

To date, there have been little data on imaging metabolism in children, in part limited by the “snapshot” methodology, the need to move unstable patients, and radiation exposure in children (137). Continuous local monitoring of basic parameters of metabolism is possible through microdialysis but has been rarely used in children. This was first described in adult TBI in the early 1990s and although clearly a useful technique for investigating brain metabolism, it has not achieved widespread utilization as a clinical tool (138). It remains a valuable tool in research-led environments, but wider adoption is likely limited by costs and effort required to run an effective program in which catheters are placed, vials changed regularly by the bedside and analyzed, and clinical decisions made on the basis of chemical changes. Little has been published in children. Tolias et al. reported a small series of children with severe TBI who underwent microdialysis monitoring, but concentrated on glutamate (139, 140). Preliminary metabolic data from microdialysis in children are in keeping with adult data (141). In this cohort of 22 children elevated lactate–pyruvate ratio was associated with mortality, poor clinical outcome, and low brain oxygen; and glucose decreased at lower CPPs.

### Management of Fluids, Hemogobin, Glucose, and Temperature

As with adults, similar controversies and uncertainties exist in the pediatric literature for fluid, hemoglobin, and glucose management, but with the confounding of having less evidence. As is usual, wide differences exist in practice at individual institutions. Normal saline is used routinely to avoid hypotonic fluids in patients with brain swelling. If CSF is being drained, sodium losses must be calculated and replaced. Some centers avoid all dextrose in fluids in the early phase of head injury, and there are variable times at which nutritional support is started (136). At our institution, we start nutrition early and do not restrict glucose in intravenous fluids but watch the systemic glucose closely. Glucose control has not been studied exhaustively in pediatric TBI. Although hyperglycemia is known to be associated with poor outcome, tight glucose control has not been studied in children with TBI. In the adult experience (142), brain glucose correlates with serum glucose, but there are discrepancies in individual patients at various times; therefore, there is presumably a greater risk of neuroglycopenia in patients who have tight serum glucose control without knowledge of brain glucose. Neuroglycopenia may be a greater problem than systemic hyperglycemia, and so caution is advised when considering tight glucose control.

Most centers use relatively conservative guidelines for initiating blood transfusion in critically ill children, and there are no specific data recommending a different practice for TBI, although the concern about brain ischemia and hypoxia is greater in these patients. We studied changes in PbtO2 before and after blood transfusion, controlling for all factors likely to influence any changes but were unable to determine any predictive factors (125). Similar results were found with transfusion for chronic anemia in children using NIRS (143). When we compared patients at the same stage post head injury, it appeared that transfusion was associated with an increase in PbtO_2_ in most but not all patients in the first few hours after transfusion, but that this difference did not persist 24 h later. One retrospective study reported that blood transfusion was independently associated with worse outcome in children with TBI (144). However, even good multivariate models cannot fully control for differences in injury severity and disease complexity that may influence the decision to transfuse. Currently, we follow guidelines for transfusion triggers in generally ill patients but raise that threshold in children who have documented evidence of cerebral ischemia or tissue hypoxia.

## Therapies

Unfortunately, there is less evidence on which to base therapies in pediatric severe TBI. Importantly, there is an ongoing multicentre comparative effectiveness trial of therapies in severe pediatric TBI with ICP monitoring that may add much needed data (145). Currently though, most recommendations are still not substantially different from the adult guidelines and are largely set at the level of an option (93). There is no evidence to support the use of hypothermia in children, despite ongoing arguments for the case in adults (146–149). Avoidance of hyperthermia, however, is widely accepted as a sound strategy. Hypertonic saline is preferred as a hyperosmolar therapy in children with severe TBI but practice is widespread, there is no standardization of use, and various formulations are used in different centers (93, 136). Long-term propofol is not used because of the concerns about fatal metabolic acidosis in children (150).

Decompressive craniectomy remains a controversial topic but the general sense is that younger patients, including children, may have greater potential benefit than in adults. Two randomized controlled trials in adults have not diminished the controversy. The DECRA trial (151) examined the use of craniectomy at a very low intervention (ICP greater than 20 mmHg for more than 15 min) in patients with diffuse injury only. The trial found that craniectomy did not benefit patients; however, their selection criteria do not generally reflect the situation in which craniectomy is commonly performed worldwide. Still it clearly established that craniectomy was not useful as a very aggressive early intervention at such a low threshold. The RESCUE ICP trial (152) included patients with mass lesions, and ICP greater than 25 mmHg for anywhere between 1 and 12 h despite stage 1 and stage 2 measures. Their results have been debated heavily since—the mortality rate was significantly lower in the surgery group but so was the occurrence of severe disability and vegetative state. Twelve months after injury and with rehabilitation, the proportion of survivors with independent function was greater in the surgical group, but the debate about what constitutes a good quality life continues. It is worth noting, however, that for ethical reasons, neither trial strictly examined craniectomy against pure medical management, as there had to be capacity for crossover. In the RESCUE ICP trial for example, almost one third of medically managed patients ended up with a craniectomy anyway.

How these results apply to children is still unanswered. The decompressive craniectomy studies in children have been single center observational trials (153–162) with the exception of one underpowered pilot study—27 children were randomized by Taylor et al. (163) and the surgery group appeared to have better outcomes. However, this was a pilot trial that was never developed further and the procedure—bilateral small temporal disk craniectomies with no dural opening—was dissimilar to the commonly employed techniques and produced only a very modest reduction in ICP. One of the problems with craniectomy in children is the high rate of bone resorption, particularly in the very young. This is further compounded by the fact that cranioplasty using foreign material is also more difficult in children with a growing skull (37). This must be evaluated with the other known complications of craniectomy including hydrocephalus (164).

## Developmental Outcomes

Better outcomes are usually reported for children compared to adults. However, there are several potential confounders in this. First, the adult cohorts often include significantly older patients, who are known to fare poorly. Second, the mechanisms and patterns of injury are different. Finally, assessment of outcome in children is made difficult by the lack of a stable baseline for comparison—children are in a developmentally accelerated phase (165). Unfortunately, various pharmacological therapies have shown promise in the laboratory but have failed to improve outcome after TBI, including most recently progesterone in the PROTECT III trial. As these trials are in adults, the data in children are limited.

Still, plasticity in children may aid recovery substantially in ways lost to adults. Enriching environments maximize this potential: animal and human studies show greater cognitive improvements associated with dendritic arborization in stimulating environments (166). That bodes well for recovery, but the developing brain is a double-edged sword. The youngest patients are at highest risk of poor outcomes because of the developmentally immature brain.

Another factor worth considering is the increasing concern about long-term inflammatory processes that may develop even after mild head injury. Arguably, the younger the age at which the injury occurs, the greater the potential for cumulative injury to occur over many years. Much of the concussion literature dedicated to understanding the long-term risk of neurodegenerative disease after mild injury concentrates on adult patients, highlighting post-mortem findings of neurofibrillary tangles and tau protein deposition (167, 168). To date, we have limited longitudinal data for children. Arguably, when the brain is injured at a young age, these changes may develop over many more years and lead to secondary neurodegenerative diseases at a much younger age than the normal population.

Even anesthesia itself may pose a risk to the developing brain. A recent warning from the FDA has increased concern and confusion about the risk of exposure to anesthetics at a young age and stirred controversy about the subject (169). The data are far from conclusive as yet, but animal studies suggest that anesthesia may induce apotosis and interfere with neuronal differentiation, synaptogenesis, and network formation, ultimately having detrimental effects on neurocognitive development (41).

## Summary

Adult clinical services underestimate differences between adults and children. There is no doubt that anatomy and physiology in children relevant to central nervous system injury is profoundly different, and there are clinically important differences even within the childhood age range. So, pediatric services must contend not only with all the uncertainties and controversies that plague the management of adult TBI but also with all the different physiologic factors in a fundamentally different and changing population for whom ironically there is much less evidence. Better treatment protocols must be developed by limiting inappropriate extrapolation from adult studies and prioritizing pediatric-specific studies to guide clinical recommendations. In absence of this, the most vulnerable population will continue to receive second-rate care because of lack of evidence.

## Author Contributions

The author confirms being the sole contributor of this work and approved it for publication.

## Conflict of Interest Statement

The author declares that the research was conducted in the absence of any commercial or financial relationships that could be construed as a potential conflict of interest.
